# Structural and Dynamic Characterizations Highlight the Deleterious Role of SULT1A1 R213H Polymorphism in Substrate Binding

**DOI:** 10.3390/ijms20246256

**Published:** 2019-12-11

**Authors:** Raju Dash, Md. Chayan Ali, Nayan Dash, Md. Abul Kalam Azad, S. M. Zahid Hosen, Md. Abdul Hannan, Il Soo Moon

**Affiliations:** 1 Department of Anatomy, Dongguk University College of Medicine, Gyeongju 38066, Korea; rajudash.bgctub@gmail.com (R.D.); hannanbau@gmail.com (M.A.H.); 2 Department of Biotechnology and Genetic Engineering, Islamic University, Kushtia 7003, Bangladesh; chayanali7@gmail.com (M.C.A.); akazadbtge@hotmail.com (M.A.K.A.); 3 Department of Computer Science and Engineering, BGC Trust University, Bangladesh, Chittagong 4381, Bangladesh; dashnayan.py@gmail.com; 4 Pancreatic Research Group, South Western Sydney Clinical School, University of New South Wales, and Ingham Institute for Applied Medical Research, Liverpool, NSW 2170, Australia; s.hosen@student.unsw.edu.au; 5 Department of Biochemistry and Molecular Biology, Bangladesh Agricultural University, Mymensingh 2202, Bangladesh

**Keywords:** binding site, cancer, molecular dynamics simulation, R213H, rs9282861, SULT1A1

## Abstract

Sulfotransferase 1A1 (SULT1A1) is responsible for catalyzing various types of endogenous and exogenous compounds. Accumulating data indicates that the polymorphism rs9282861 (R213H) is responsible for inefficient enzymatic activity and associated with cancer progression. To characterize the detailed functional consequences of this mutation behind the loss-of-function of SULT1A1, the present study deployed molecular dynamics simulation to get insights into changes in the conformation and binding energy. The dynamics scenario of SULT1A1 in both wild and mutated types as well as with and without ligand showed that R213H induced local conformational changes, especially in the substrate-binding loop rather than impairing overall stability of the protein structure. The higher conformational changes were observed in the loop3 (residues, 235–263), turning loop conformation to A-helix and B-bridge, which ultimately disrupted the plasticity of the active site. This alteration reduced the binding site volume and hydrophobicity to decrease the binding affinity of the enzyme to substrates, which was highlighted by the MM-PBSA binding energy analysis. These findings highlight the key insights of structural consequences caused by R213H mutation, which would enrich the understanding regarding the role of SULT1A1 mutation in cancer development and also xenobiotics management to individuals in the different treatment stages.

## 1. Introduction

Various endogenous and exogenous compounds in the body are metabolized by the sulfonation process, which is generally initiated by sulfotransferases [1,2]. Among the members in the sulfotransferase family, SULT1 isoform SULT1A1 (Sulfotransferase under the Family of 1A Member 1) is the most abundant and distributed throughout different tissues [3,4]. Compounds including, drugs, steroid hormones, neurotransmitters, heterocyclic aromatic amines (HAAs), and some polyaromatic hydrocarbons (PAHs) [4,5,6,7,8] generally undergoes the sulfonation process [9,10], where the addition of sulfonate (SO_3_^−^) group enhances the substrate hydrophilic potentiality or polarity and generates less toxic metabolites, and finally facilitates them for excretion [5,11]. In some cases, sulfonation of exogenous compounds turns them into active mutagens and carcinogens [12,13,14], although it sometimes acts as a chemical defense mechanism against xenobiotics [15].

Recently, mutation at 213 positions of SULT1A1 exon 7 (rs9282861) [16,17] was reported by multiple studies to be involved in breast cancer progression [18], especially among the postmenopausal Asian women [19,20]. A meta-analysis showed that polymorphic rs9282861 has a significant association with bladder cancer [21], while a small scale case-control study found a significant association with the prevalence of intestinal cancer [22]. The significant association was also found for developing head and neck cancer [23], lung cancer [11,17,24], and also accountable for acute leukemia in infants and acute myeloid leukemia [25]. Patients having rs9282861 mutation, which caused the change of an amino acid from arginine to histidine at the 213th position (R213H), may have a higher susceptibility for developing esophageal cancer, due to the lower enzymatic activity and thermal stability that affect the procarcinogens metabolisms [4,8,26,27], including various steroid hormones, neurotransmitters, and non-opiateanalgesics [28,29]. Furthermore, the substitution occurred with amino acid having the same side chain polarity, although the mutation was reported to reduce the binding affinity of various substrates, as well as their pharmacokinetic properties [27,30,31].

Despite the pathological role of this rs9282861 mutation in SULT1A1, the contribution of this substitution in the alteration of substrate-binding pocket and reduction in enzymatic activity is not clearly understood and need to be clarified in detail at molecular levels. In the present study, we performed a detailed analysis of the role of SULT1A1 R213H in substrate binding by using long-term molecular dynamics simulations. Here we show the atomic conduct of conformational change in regard to destabilization, secondary spatial structures and folding schemes of wild and R213H SULT1A1.

## 2. Results

### 2.1. Architecture of SULT1A1 and Stability of Simulation Systems

**Figure 1:**
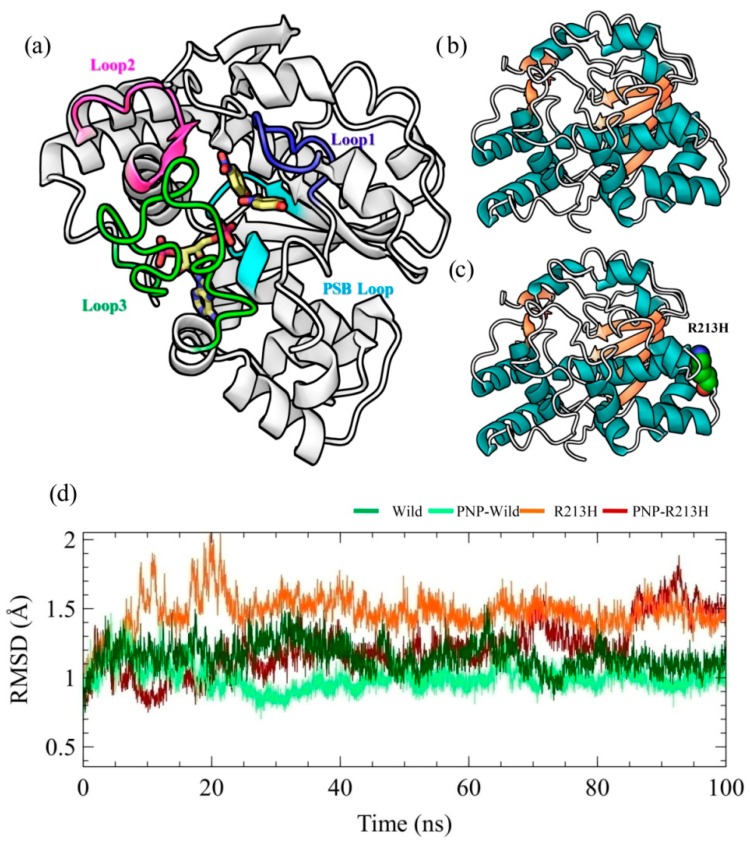
Substrate binding motifs in SULT1A1 (**a**), along with wild (**b**) and mutated (R213H) (**c**) type structures. Protein thermodynamics stability during the simulation was evaluated through root-mean-square deviations (RMSDs) for wild, R213H, PNP-Wild complex, and PNP-R213H complex, by considering backbone atoms (C, Cα, and N) of protein (**d**). Here, dark green, light green, orange, and dark red colors denote wild, PNP-Wild, R213H, and PNP-R213H, respectively.

The SULT1A1 is a sphere-shaped protein, which has a single α/β domain and α-helix surrounded five β-sheets at the central region (Figure 1a). The β-sheets are responsible for the substrate binding and are conserved in cytosolic sulfotransferase [32]. The dimerization site relies on the N terminal end of sulfotransferase and hypothesized as the source of substrate inhibition [33]. The catalytic activity of SULT1A1 depends on the Glu^83^, Asp^134^, Glu^246^, and Asp^263^ residues [2,34]. The mentioned structure contains a connected molecule named *p*-nitrophenol (PNP). The motif, ^257^RKGMAGDWKXXFT^269^, which is similar to the P-loop observed in ATP and GTP binding proteins, is supposedly essential for the PAPS binding. The 5′-phosphate positioning is important for the organizing co-factor, thus transferring sulfonate group to the substrate [5,17]. The ^45^TYPKSGTT^52^ loop is termed as phosphosulfate binding (PSB) loops of SULT1A1 and responsible for binding 5′-phosphate group of PAP. There are three substrate binding loops of SULT1A1 named as Loop1 to Loop3 with residues of 81–90, 145–154, and 235–263, respectively. The substrate binding site of SULT1A1 is hydrophobic and flexible, permitting this enzyme to embrace various structures, thus it can interact with a variety of L-shaped uncharged substrates including, aromatics (diiodothyronine), tiny aromatics (PNP), and fused ring compounds [2,31,35,36]. The mutation of our interest, R213H is located between Loop2 and 3, and its contribution to SULT1A1 dysfunction was systematically analyzed by 100 ns molecular dynamics simulation by considering both wild and mutant type structures in the presence and absence of substrate (Figure 1b,c).

The root-mean-square deviation (RMSD) of the protein was analyzed in all systems for the original description of simulation performance and stability, reflecting the general thermodynamic stability of the system (Figure 1d) [37]. The RMSD represents the conformational stability of the protein structure during the course of the simulation, where the larger deviations indicate more flexibility of the protein structure [38]. According to the RMSD plot, all wild and mutant type structures with and without ligand achieved equilibrium after 10 ns and remained stable afterward, which allows an appropriate framework for further analyses [39,40]. In both wild and mutant types, conformational flexibility of the SULT1A1 was seen to reduce by the binding of ligand, as revealed from the RMSD calculation (Figure 1d). Although it has been ascertained that many proteins undergo side-chain flexibilities upon the binding of ligands [41], the findings derived from RMSD calculation are consistent with the previous experimental results that ligand binding decreases backbone flexibility of SULT1A1 [42,43,44,45,46]. Furthermore, the RMSD patterns were changed due to the mutation in both apo and holo form, indicating that mutation induced the changes in overall structure, in terms of flexibility and ligand stability.

### 2.2. Effects of Mutation on Conformation Stability

**Figure 2:**
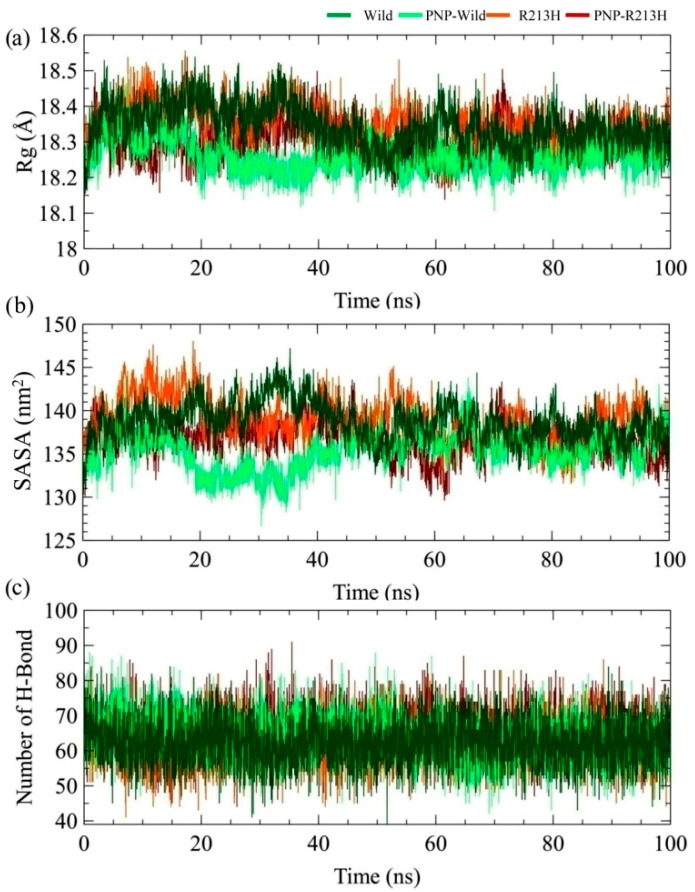
Stability of the protein during the simulation by means of radius of gyration (**a**), solvent accessible surface area (SASA; (**b**)), and number of intra-residue hydrogen bond (**c**) for both wild and mutated type structures. Here, dark green, light green, orange, and dark red colors denotes wild, PNP-Wild, R213H, and PNP-R213H, respectively.

To observe the outcome of the mutation on inclusive protein stability, the radius of gyration (Rg) was calculated, which indicates the mean-square mass-weighted root range of a set of atoms that shared the mass centre [47]. The higher Rg values indicate loose packing of the protein structure, which means conformation that is more flexible. Additionally, the solvent accessible surface area (SASA) was calculated, where the lower values of SASA indicates the contracted nature of protein [48]. According to the Rg analysis, no significant difference was found between the wild and mutated protein, and a similar effect was also observed in case of SASA profile (Figure 2). However, binding of the ligand showed the effect in protein conformation, where the mutated PNP-R213H complex showed the higher Rg value for around 18.13–18.5 Å and the lower Rg value was observed for the PNP-Wild complex around 18.1–18.48 Å (Figure 2a). In the case of the wild type, it was clearly seen that the flexibility of the protein was reduced after binding of the ligand, thus increasing the compactness of protein folding. In contrast, the mutant type retained similar flexibility patterns to its apo form. The similar fashion of structural deviation in wild and mutant types was also observed in the SASA calculation (Figure 2b), where binding of ligand reduced the total SASA values. The PNP-R213H complex showed a higher SASA value than the wild type complex. At the initial stage, R213H complex was seen to fluctuate but after 7 ns, it remained stable and continued to 50 ns. Afterward, the SASA values decreased rapidly at 60 ns and again remained stable at 70 ns to the rest of the simulation period.

The PNP-Wild complex, on the other side, showed less SASA values about 126.5 nm^2^ at 31 ns and its retained stability at 40 ns, and also persisted stable through the simulations. The overall flexibility of the protein can be modulated by the number of the internal hydrogen bond, which was calculated and shown in Figure 2c. However, no significant difference was observed in the total hydrogen bonding (Table S1). Overall, Rg and SASA calculation denoted the mutation-induced flexibility in the protein–ligand complex, which was consistent with the RMSD analysis.

### 2.3. Effects of Mutation in Protein Dynamics

**Figure 3:**
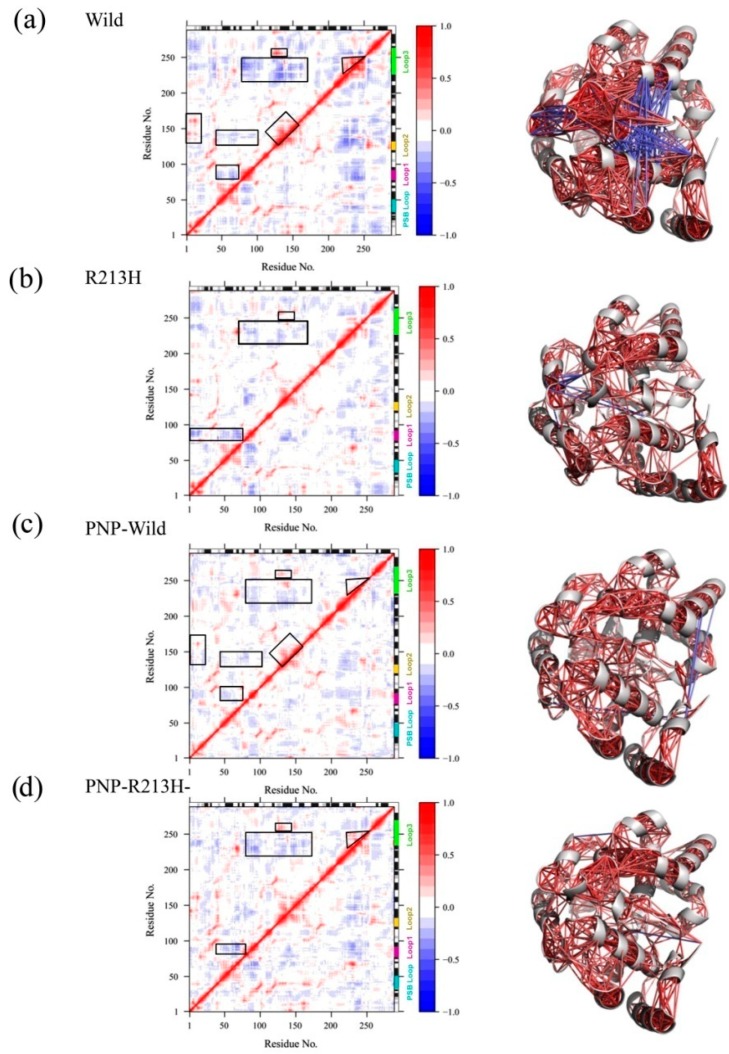
The residual correlative motion in protein structures was visualized by the dynamic cross correlation map analysis, for wild (**a**), PNP-Wild (**b**), R213H (**c**), and PNP-R213H (**d**). Here, strong correlative motions (±4 to ±1) were depicted in three dimensional cartoon model on the right side of each panel. In all cases, correlations were represented in the color spectrum, from red to blue. The positive correlation between two residues, that is, both residues move the same direction, is represented as red color. On the other hand, residues having opposite motion are denoted as the anti-correlative motion, marked as blue color.

Correlative motions play a crucial part in recognizing and interacting bio-macromolecular system, which can be achieved from the covariance equation of molecular fluctuation generated through the molecular dynamics (MD) simulation trajectory. The correlative motions of different simulation systems were analyzed by the dynamic cross correlation matrix (DCCM). The DCCM fluctuations are depicted in Figure 3, which graphically displays time-correlated information among the residues of the protein. The deeper color intensity highlights more positive or negative correlated motions between the structures. The red color indicates strong positive correlation whereas; the blue color indicates a negative correlation. The R213H protein and PNP-R213H complex exhibited different movements compared to wild protein and PNP-Wild complex, respectively. Higher positive and negative correlations were observed in wild type, especially within the inter loop residues of the active site, that is, between loop3 and loop2 (Figure 3a). Loop3 also showed negative correlation with the residues located in loop1, however, residues contained in the loop1 were further anti-correlated with the PSB loops containing residues. Besides, the mutation showed only the negative, but insignificant, correlations between the residues of PSB loop and loop1 (Figure 3b). Interestingly, these correlations were lost in the case of ligand binding conferring the stable protein-ligand complex; instead, the complex generated random motions within the loops (Figure 3c). The ligand bounded R213H protein exhibited the same correlations as in unbounded forms. Moreover, a few but new types of correlations were observed between loop1 and loop3 (Figure 3d), which was different from the wild complex.

**Figure 4:**
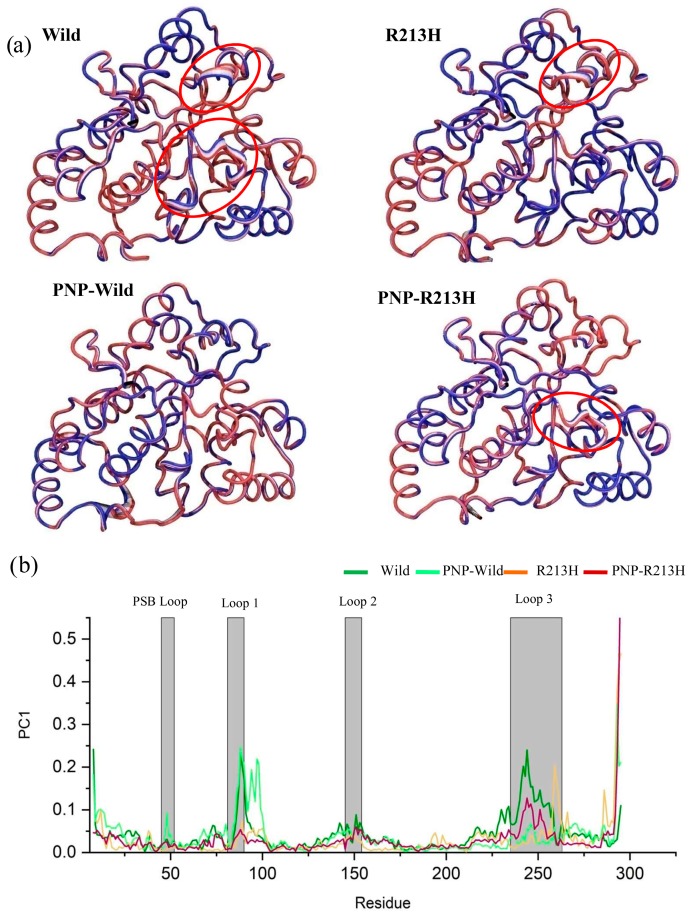
Changes of protein dynamics due to mutation by means of principle component analysis. Here, principle component 1 is represented in the tube structures of the three dimensional structure of wild, PNP-Wild, R213H, and PNP-R213H, respectively (**a**). The widen tube means flexibility, highlighted by the red circle. In addition, residue based mobility plot showing the variation in the mobility in PC1 for different simulation systems, where dark green, light green, orange, and dark red colors denote wild, PNP-Wild, R213H, and PNP-R213H, respectively (**b**).

In addition, principle component analysis (PCA) was performed to observe whether these correlations resulted in any significant flexibility of the protein. The PCA can be applied to any system and permits to study the influence of any varying parameters, by lessening the collective motions complexity [49,50,51,52], which is associated with the phase space behavior related to protein functions and stability. Therefore, it is frequently used to characterize the conformational variances involved in the protein folding [53,54], opening and closing mechanisms of ion channels and other proteins [55,56,57,58], as well as the conformational dynamics induced by the mutations [46,50,59,60] and the ligand binding [61]. The motion of the C_α_ atoms was described by the eigenvectors of the covariance matrix, which were calculated in PCA analysis and resulted in first PCA of 22.72%, 23.03%, 16.48%, and 21.05% for wild, R213H, wild-PNP, and R213H-PNP systems, respectively (Figure S1). Compared to the wild and wild-PNP systems, the apo and holo structure in the mutated form showed unusual patterns on to the phase space of the foremost two principal components (PC1 and PC2), describing the significant alternation in the protein conformation. Furthermore, the highest PC1 in the wild apo structure described that wild apo structure was undergone conformational changes, while its ligand binding state, i.e., binding with ligand reduced the conformational stability (Figure 4a). A closure looks to the local residue mobility, in terms of PC1, ensured that the conformational changes were induced in the active site loops, which was decreased binding of ligand and in mutated forms (Figure 4b). In mutated form, the holo structure increased the movement in loop3, while reduced in PSB loop and loop1 and 2, compared to both wild type structures (Figure 4b). These results are correlated with the finding from DCCM analysis and confirmed that the residues in the active site endured conformational changes due to the mutation.

**Figure 5:**
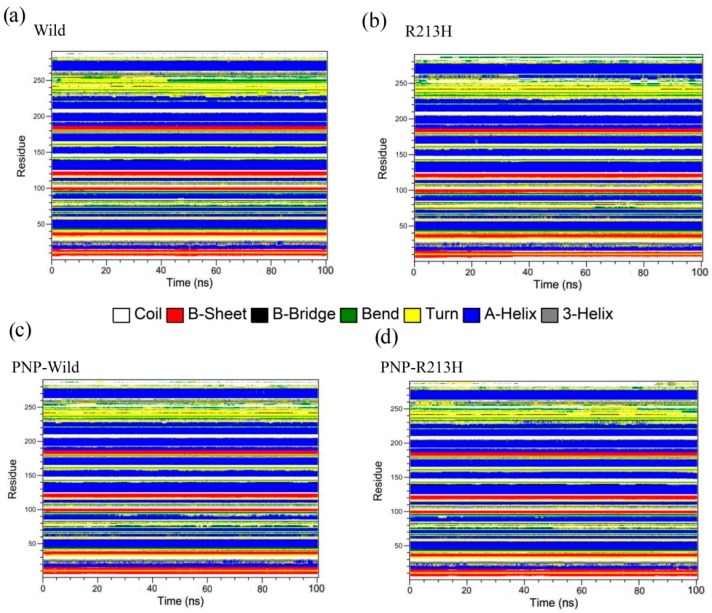
Evolution of the secondary structure elements of wild (**a**), R213H (**b**), PNP-Wild (**c**), and PNP-R213H (**d**) during molecular dynamics simulations based on DSSP classification.

With the purpose of observing the dynamics changes in the active site, Define Secondary Structure of Proteins (DSSP) analysis was performed to analyze the structural stability in the loops, shown in Figure 5, which provides detailed information about the loss and gain of the secondary structure all through the simulation. As shown in the results, the secondary structure content was stably maintained by all residues in both wild and mutant types, except the residues located in the loop3. The residues located 235–263, showed diverse conformations throughout the simulation in different systems. In wild type, these residues, including 255–263, showed 3-helix conformation over time (Figure S2). However, this conformation was changed to A-helix after 20 ns of simulation due to the mutation (Figure S2c). Furthermore, mutation reduced the bend conformation of the loops, which seemed to increase in case of wild complex structure (Figure S2c). In both apo and holo structures, mutation induced the persistency of B-bridge conformation in the loop3 (Figure S2) denoting that mutation increased the rigidity of the loops that may damage the plasticity of the active site.

### 2.4. Effects of Mutation in Active Site and Ligand Binding

**Figure 6:**
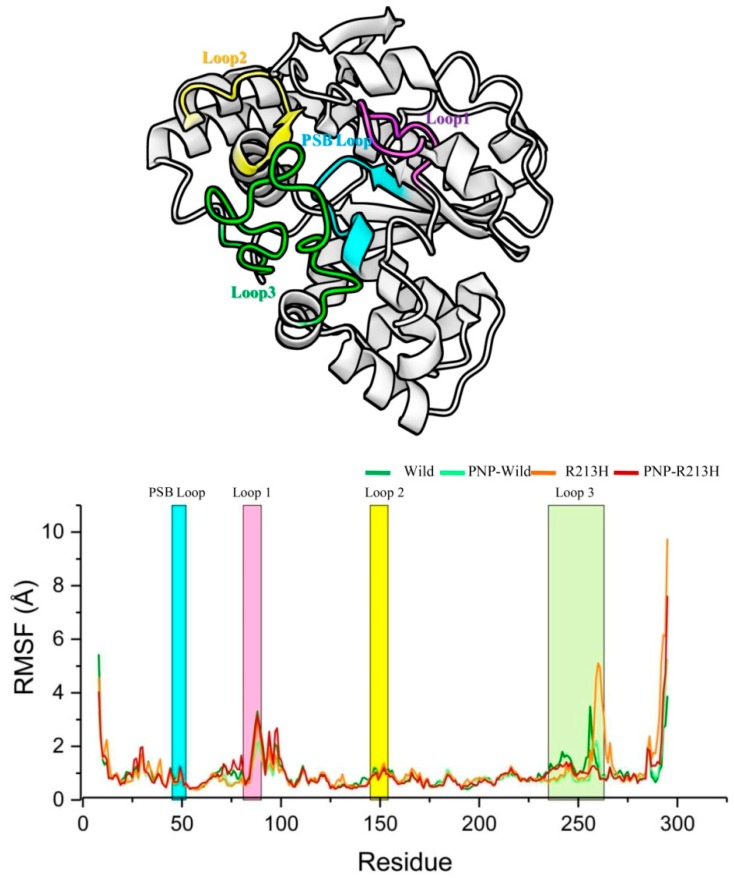
The root mean square fluctuation (RMSF) of the Cα atoms for the wild protein, R213H, PNP-Wild complex, and PNP-R213H complex. In this graph, dark green line represents wild protein, whereas light green, orange and dark red colors denote wild, PNP-Wild, R213H, and PNP-R213H, respectively. The horizontal *x*-axis bar region denotes different loop regions.

Before the detailed investigation, the residue fluctuation profile due to the mutation was considered, thus RMSF analysis was performed on simulation trajectories (Figure 6). The RMSF calculation represents the dynamics of the backbone atoms, where higher values designate greater flexibility through the MD simulation. The highest peaks on this graph show the protein regions, which were mostly fluctuated throughout the simulation period. The protein in all simulation systems showed RMSF within a range of 0.2–9.8 Å. As can be seen in Figure 6, the overall RMSF value of the residues of wild and mutated proteins was almost the same in the PSB loop, loop1, and loop2, however, the difference was observed in loop3, indicating a major binding site of the ligand. Evidently, R213H mutation induced higher fluctuation in the loop3 segment compared to the wild type, which was reduced by ligand binding. For instant, binding of ligand showed less fluctuation compared to wild type, while the mutation reduced more than that of wild type.

**Figure 7:**
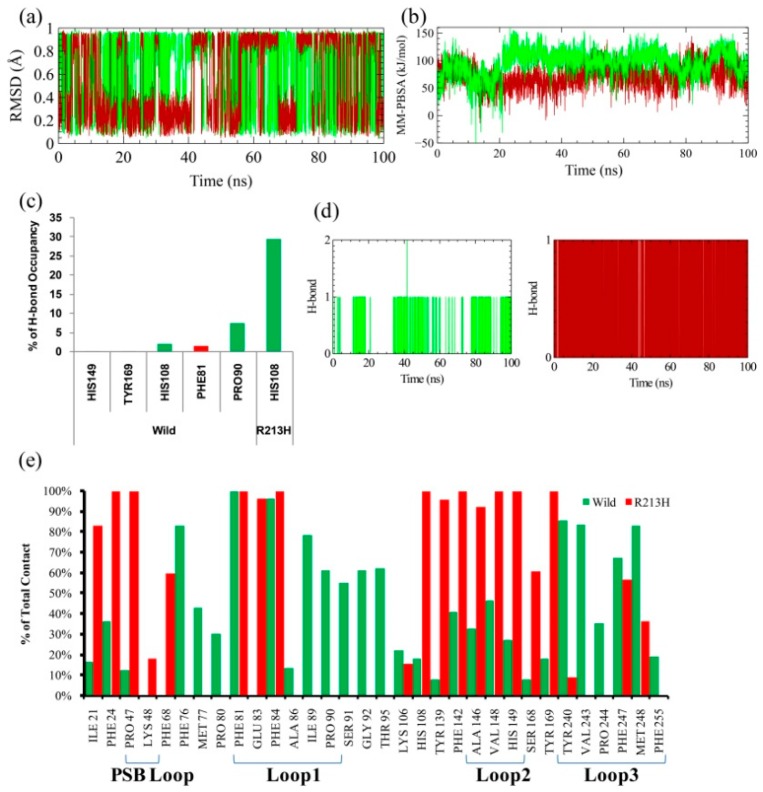
Ligand RMSD in both wild (green line) and mutated (dark red line), which means the degree of ligand flexibility in substrate binding site (**a**). MM-PBSA binding energy analysis calculated from the trajectory of 100 ns molecular dynamics simulation with AMBER14 force field, using YASARA dynamics software (**b**). The total number of intermolecular hydrogen bonds (**c**) and their occupancy with their respective donor and acceptor (**d**). Total intermolecular contact analysis (**e**). Here, green color represents the residues of wild protein with their contact percentage where red color indicates R213H protein contact percentage.

Accordingly, the ligand RMSD and intermolecular interaction profiling was considered to analyze the effect of rigid behavior of loop3 on ligand binding, where the plot showed that mutation reduced the ligand flexibility in the active site, while ligand was seemed to be very flexible in the pocket of native structure, as revealed from the wild type complex (Figure 7a). Likewise, the intermolecular hydrogen bonding analysis showed that the number of hydrogen bonding partners, specifically, residues from the active site was decreased in the mutated structure; the compound only showed maximum interaction with His108 (Figure 7c). Surprisingly, mutation also reduced the total contact with the active site residue (Figure 7e), and induced a notable change in protein–ligand interaction network. Specifically, the total number of ligand-mediated contact was reduced in the mutant complex in the region of loop1 and loop3 (Figure 7e), however, mutation induced the total contact with the residues in loop2, including Ala146, Val148, and His149. Besides the ligand also maintained the contact with the residues from PSB loop such as Pro47 and Lys48, whether these inter molecular contacts were favorable to binding affinity or not, additionally binding energy calculated by MM-PBSA method was conducted on resulted 4000 snapshots from 100 ns simulation [62]. MM-PBSA binding energy results were depicted against time for wild-PNP and PNP-R213H, which is shown in Figure 7b. The average binding energy for wild-PNP complex was 110 kJ/mol, while 60 kJ/mol for mutant types (Table 1). Previous studies based on biochemical pharmacogenetic assays presented that, R213H caused very low thermostable sulfonation activity to 4 mM 4-nitrophenol, while the wild type exerted a higher binding affinity [63,64]. Consistent with previous findings, the MM-PBSA analysis thus revealed that the mutation reduced the binding affinity to the PNP, as a result of changing intermolecular contacts, which might contribute to low sulfonation activity.

### 2.5. Insights Into Substrate Binding

**Figure 8:**
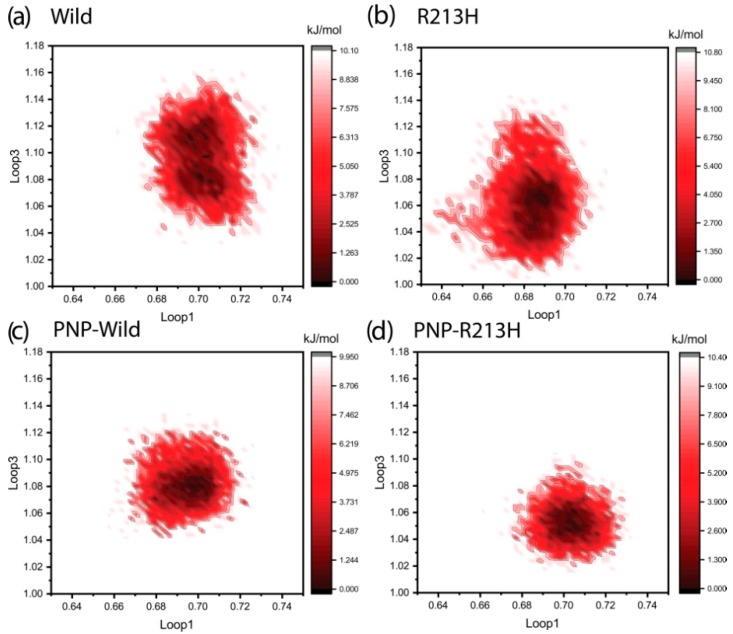
Energy landscapes of different simulation systems including Wild (**a**), R213H (**b**), PNP-Wild (**c**), and PNP-R213H (**d**) respectively. Here the reaction coordinates are the Rg (nm) of loop1 (residue 81–90) and loop3 (residues 235–263) as x and y axis, respectively. The color scheme to the right of each plot is given in units of kJ/mol.

Since the mutation disrupted significant intermolecular interactions between the loop residues and the ligand, the conformational variability of these loops was mapped onto the free energy landscape by considering the radius of gyration of loop1 and loop3 as the reaction coordinates. The disperse area with color depth from light to dark red color indicates the conformational variability of the loops, where the darker area represents the conformation with the lowest energy minima. The degree of dispersion describes the conformational flexibility, where the larger area denotes more flexibility. As can be seen in the Figure 8, the conformation of the loops with the lowest energy was dispersed through the projection, where the two largest resulting clusters were seen to shift towards the larger Rg value, describing the loops flexibilities during the simulation (Figure 8a). On the other hand, the energy minima of R213H was seen to shift towards the lower Rg values, in the case of both loops, demonstrating the loss of loop flexibility (Figure 8b). In the case of ligand binding, wild type showed normal distribution in the phase behavior, moreover concentrated, which explains loop contribution to the ligand binding (Figure 8c). Consequently, mutant reduced the conformation flexibility more than the wild type in case of ligand binding, where the flexibility of loop3 was seen to be reduced, while the distribution energy minima were shifted toward the larger value of loop1 (Figure 8d).

In order to understand the effect of mutation in the substrate binding, the conformer having the lowest energy was further isolated from the simulation trajectories of holo protein based on the additional free energy landscape analysis. The free energy landscape was thus constructed based on the RMSD and Rg time-dependent changes to the original structure, which demonstrates the effect of mutation on global conformational space of SULT1A1 (Figure S3). The energy minima in the figure were represented from red to blue color, where the more concentrated blue zones described the more stable conformation with global minimum energy. On the other hand, the shape and size of the area display the conformational stability of the protein. As can be seen in Figure S3, the distribution of global energy minima was changed due to the mutation, although both wild and mutant types exhibited Gibbs free energy varying from 0 to 11 kcal/mol. Since SULT1A1 in both wild and mutant types exhibited stable conformation in the lowest energy level of 0 kcal/mol, the respective conformation was visualized and analyzed for intermolecular interaction analysis (Figure S3).

**Figure 9:**
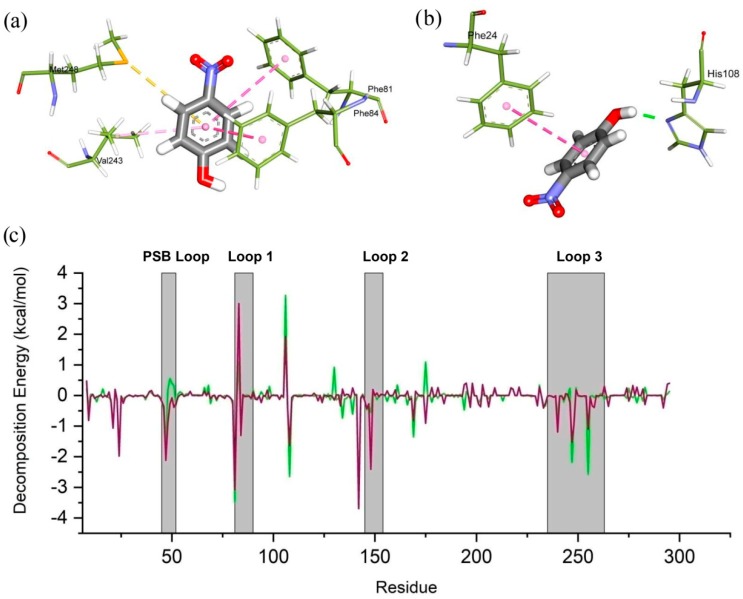
The intermolecular non bonded interaction pattern is rendered for wild (**a**) and mutated (**b**) complex derived from the free energy landscape. Here, pink color denotes Pi-alkyl bond, yellow for pi-cation, and green for hydrogen bond, respectively. Per-residue decomposition analysis (**c**). Here, green color denotes PNP-Wild protein whereas, magenta color represents PNP-R213H protein.

Interestingly, significant fluctuations were noted between wild and mutant forms in the protein–ligand interactions (Figure 9). In wild complex, the ligand formed multiple hydrophobic interactions with the residues from loop1 and loop3, including Phe81, Phe84, Val243, and Met248 (Figure 9a). On the other hand, it showed only hydrophobic interaction with Phe24 residue and hydrogen bond with His108 in case of mutant type (Figure 9b), which indicates that mutation might alter the active site of the protein, therefore the volume and total SASA of the ligand binding pocket was calculated with the help of CASTp server, where the higher SASA is more pronounced for hydrophilicity, shown in Table 1. The analysis displayed that mutation increased the active site volume and SASA of apo SULT1A1, however, the volume was reduced in complex with ligand, in both cases, SASA was still increased (Table 1).

Moreover, per-residue decomposition analysis, which describes the contribution of each resides in total binding energy, was conducted to elucidate the mutation-induced effect on ligand binding, based on the supplementary 25 ns MD simulation by GROMACS software. Noticeably, the mutation caused significant changes in the binding energy contribution, where it was represented that, mutation reduced the favorable contribution of the residues from loop1 and loop3, especially Phe247, Phe255 and Phe81, and Phe84 respectively, while it increased the unfavorable contributions of Leu82, Glue83, Ala86, and Tyr240 (Figure 8c). Furthermore, the mutation also showed a reduction in the total MM-PBSA binding energy than the wild type, supporting the previous MM-PBSA analysis by YASARA dynamics calculation. These interpretations were consistent with fluctuated notion that conformational flexibility of loops in the active site plays a vital role in the ligand binding, which eventually disrupted by R213H.

## 3. Discussion

Sulfonation has been recognized as the vital metabolic reaction for metabolizing not only various endogenous compounds but also bioinactivation of a variety of xenobiotics [10,65,66,67] through the action of cytosolic sulfotransferase (SULT). The genetic polymorphisms of *SULT1A1* [26,68,69,70] caused the theatrical changes in the protein function. In this study, we considered SULT1A1 R213H, which is associated with various cancers. To look for key structural consequences offered by the mutation for this deleterious behavior, a series of comparative analyses were accumulated in this study based on computational analysis.

The molecular dynamics simulation was thus conducted to reveal the dynamic changes of the protein due to the mutation in both apo and holo form, where the initial characterization by RMSD showed that mutations induced different transitional pathways from the initial to the final stage of simulation. Consistent with the RMSD profile, the Rg and SASA calculations also confirmed the mutation induced conformational flexibility in both apo and holo form of SULTA1, where mutated types increase the dimension and solvent accessibility of the protein. In dynamics condition, protein undergoes various conformational changes for particular function, where residual communication in terms of correlative motions serves a vital role in recognizing and binding of various biological macromolecules, and this communication is usually disrupted by the mutation [71]. The correlative motion in SULTA1 based on the DCCM analysis showed that mutation reduced the correlative motions particularly in the loop1 and loop3 of active site as a results structure flexibility is lost in those positions, which was clearly reflected through collective motion analysis by PCA. Furthermore, mutation also increased the correlated motions in mutated-PNP complex compared to the other systems, which indicates the functional disruption of SULTA1. These consequences were further supported by RMSF analysis, where the effect was revealed in the loop1 and loop3 regions.

The active site of SULT1A1 is plastic, which is maintained by the aforementioned three loops, can process various conformational changes to adopt a verity of aromatic compounds [2,31,35,36]. Furthermore, the binding of these aromatic substances to the active site is highly modulated by Phe142 and Phe81, which creates an entry portal that allows only the catalytic site to bind linear substrates [72]. On the other hand, Val148, Phe247, and Met248 in the loop3 maintained interactions with nitrogen containing groups of the substrate followed by water bridge and van der Waals bonds [31].

Previous study showed that substitution of His213 makes the protein more thermolabile than the wild type [73], where the crystal studies presented that the mutation influence both structural stability and substrate regulation of SULT1A1 [31]. In this regard, Lu and colleagues identified that the substrate binding affinity and kinetics are susceptible to the loss of secondary structures stability [74], which is achieved by the mutation. Perhaps, it has been presumed that the mutation might influence the PAPS binding to PSB loop by changing the interaction of Tyr62-Arg213 [30], thus contribute to loss of enzymatic activity. Interestingly, total contact analysis showed that mutation reduced total number of interactions with the ligand during the simulation, which was also revealed in the analysis of the most native structure with lowest energy minima, calculated from the free energy landscape. Furthermore, it appeared that the active site volume and total hydrophobicity were reduced (as SASA is increased) due to the mutation; thus, the ligand flexibility in the active site was hindered, which was necessary for maintaining maximal interactions with the pocket residues. Besides, the increase of hydrophilicity in the active site resulted in unfavorable binding of the ligand, which might affect ligand binding properties as well as selectivity.

Overall, the PAPS site of cytosolic SULTs is characterized by the conserved residues, however, the core hydrophobic substrate binding site exhibits wide range of substrate specificity, which allows sulfonation for a specific substrate [2]. Accordingly, the uncharged phenolic compounds are preferred by SULT1A1, where the mutation R213H disorders the plasticity of substrates binding loops and increased the hydrophilicity of the pocket, affecting the sulfonation process, and thus contributes in cancer progression.

## 4. Materials and Methods

### 4.1. Preparation of the Simulation System

The structure of SULT1A1 has been extracted from the protein database (http://www.rcsb.org/pdb) [75] in three-dimensional crystal frameworks with a protein data bank (PDB) ID of 1LS6 [31], having the molecular mass of 34.165 kDa. The structure was initially prepared by adding bond orders, hydrogen and charges; and also refined by removing water molecules and optimizing it at neutral pH. The structure was further fixed by correcting some amine derivatives, thiol groups, hydroxyl groups, protonated glutamic acids, aspartic acids, and histidines. In order to adjust the heavy atom of the structure, energy minimization has been applied by using optimized potentials for liquid simulation (OLPS3) force field until the RMSD reached to 0.30 Å. Since the structure already contained the histidine residue at the position of 213, the wild type structure (H213R) was further constructed by computational mutagenesis methodology, using Schrödinger Mutate Residues script 2017-1 suite (LLC, New York, NY, USA) [76]. Additional refinement by short molecular dynamics simulation was performed to improve the structure quality. For that, 500 ps of molecular dynamics simulation was conducted by using Yet Another Model Building and Energy Refinement force field (YAMBER3) force field [77], followed by maintaining 298 K temperature at pH 7.4. The simulation was performed in the transferable intermolecular potential3 points (TIP3P) water model with a density of 0.997. Based on the lowest energy, the final representative structure was considered for further study. For ligand binding characterization, the native co-crystal ligand, *p*-nitrophenol (PNP) was only considered and kept in protein structure. Accordingly, four individual simulation systems were developed, including wild, wild-PNP, R213H, and R213H-PNP.

### 4.2. Molecular Dynamics Simulation

The consequence of mutation on SULT1A1 protein structure and stability was analyzed by molecular dynamics simulation. The YASARA dynamic software (YASARA Biosciences GmBH, Vienna, Austria) [78] was used to analyze the dynamics of the wild and R213H type structure in the presence and absence of ligand, according to the previous protocols [59,62,79,80]. The process was carried out by cleaning each structure, followed by optimizing the hydrogen bond network. A cubic simulation cell was then constructed with a periodic boundary condition. The assisted model building with energy refinement14 (AMBER14) force field [78,81,82] was used to input the atoms of every single complex. This force field was preferred because, in regards to knowledge-based interactive potentials of the homology models, it was optimized for structure prediction, refinement, and energy minimization. The ligand was parameterized by AutoSMILES [83] algorithm, which used combined, AM1BCC [84] and General AMBER Force Field (GAFF) [81] for typing atomic charges and bond orders to unknown organic molecules. The TIP3P solvation system was used with 0.997 g/L density for solvating the simulation cell, and acid dissociation constant values (pKa) were calculated for titratable amino acids in the protein [85]. The TIP3P solvent system was known to give the best experimental output with a combination of AMBER14 force field [86,87,88,89]. The pH of the system was maintained at 7.4 to mimic the physiological conditions, and accordingly, the protonation states of each amino acid residue were determined in combination with H-bonding network and SCWRL algorithm, which employs dead-end elimination and graph theory [90]. Furthermore, the solvation system was supplemented with Na^+^ and Cl^−^ ions [91]. Followed by energy minimization through the steepest descent approach, the conformational stress of the system was reduced by applying simulated annealing protocol. In order to explain the long-range electrostatic interactions, the Ewald particle mesh (PME) was used, where the distance cut-off was set to 8 Å. Finally, 100 ns molecular dynamics simulation was performed with Berendsen thermostat at a time step interval of 2.00 fs, together with a multiple time step algorithm [92,93]. The pressure was set constant and trajectories were accumulated with an interval of 25 ps. A preinstalled macro (md_run.mcr) within the YASARA suite was used for all simulation phases, while the results was characterized using YASARA suite along with VMD (Version 1.9.3, Theoretical and Computational Biophysics Group, Urbana, IL, USA, 2016) [94] and DSSP (EMBL, Heidelberg, Germany) [95,96] tools. The corresponding trajectories were used in different evaluative procedures to analyze structural insights and stability through root mean square deviation (RMSD), radius of gyration (Rg), solvent accessible surface area (SASA), number of hydrogen bonds, dynamic cross correlation matrix (DCCM), root mean square fluctuation (RMSF), and secondary structure element (SSE) analysis.

Initially, the resultant trajectories were subjected to evaluate binding energy by MM-PBSA calculation, for that integral YASARA binging energy Macro was used. The method of calculation follows the theory of nuclear physics [97]; that is, the better binding is indicated by positive energy [62,98]. The equation is as follows,

Binding Energy = E_potRecept_ + E_solvRecept_ + E_potLigand_ + E_solvLigand_ − E_potComplex_ − E_solvComplex_ (1)

where, YASARA built in Macros was used to calculate MM-PBSA binding energy, using AMBER 14 as a force field, where the more positive energies indicate the better binding [99]. The approach is similar to MM/PBSA, reported by [100].

### 4.3. Dynamic Cross-Correlation Map (DCCM) Analysis

The Bio3D [101] package integrated with R program was used to calculate residue-residue dynamic cross-correlations to determine how mutations influence the inner dynamics of protein conformations. The Cα-coefficient of cross-correlation was calculated by the average structure. Bio3D “DCCM” derives co-variation of the matrices internally from the coordinates provided and calculates Pearson’s co-variance matrices correlation coefficients, calling on “cov2dccm”. Based on the following equation, a cross-correlation ratio, C_ij_, has been considered for the Cα electrons [102]:

(2) $$C_{\mathrm{ij}}=\frac{\left\langle \Delta r_{i}\cdot \Delta r_{j}\right\rangle }{{\left\{{\left\langle \Delta r_{i}\right\rangle }^{2}{\left\langle \Delta r_{i}\right\rangle }^{2}\right\}}^{1/2}}$$

where, the average location of i^th^ and j^th^ residues were represented by Δr_i_ and Δr_j_. The time average was represented by the angular bracket. The DCCM values were between − 1 and + 1, where the positive correlation was represented by the positive value and negative correlation represented by negative values.

### 4.4. Principal Component Analysis (PCA)

Generally, PCA is used to understand the dynamics of any biological system. This technique has been used to achieve diagonal eigenvectors and the equivalent eigenvalues based on the calculation and diagonalization of the covariant atomic fluctuation equation [103,104]. This is also called by their eigenvectors are principal components (PCs), which supply data with their corresponding vectors on movement and displacement of the atoms. The mathematical details have been described previously [105,106].

### 4.5. Free Energy Landscape Analysis

The free energy landscape maps all the probable macromolecular conformational modifications, by emphasizing their respective energy levels to detect the location of the interaction molecules in the system [107,108]. In the evaluation of free energy landscape, the stability of protein was defined by Gibb’s free energy calculation. It also represents the various structural nature of protein’s structure-function correlation. In this study, the wild, and mutant protein free energy landscapes were analyzed by the following equations:

G_i_ = − K_B_Tln (N_i_/N_max_) (3)

where, Boltzmann’s constant is signified through K_B_, T stands for temperature (300 K), population bin I is presented by N_i_ and the most inhabited population bin was depicted by N_max_. As the smallest provability, an unnatural barrier scale was set for the bin without any populations. A color code model was used to display different energy levels.

### 4.6. Per Residue Energy Decomposition Analysis

In order to reveal the contribution of active site residue in the binding energy in both wild and mutated system, per residue energy decomposition analysis was performed by using the g_mmpbsa tool, as a function of APBS and GROMACS, thus independent MD simulations were individually performed for each of the complexes [109]. The lowest energy structures of protein–ligand complex derived from the free energy landscape analysis in both wild and mutated type were subjected for additional 25 ns molecular dynamics simulation by GROMACS software, according to the previously described method by Kumari et al. [109], where the protein was parameterized by the AMBER ff99SB force field [110], and the AM1-BCC and GAFF were considered for the van der Waals and bonded parameters for the ligand (See Supplementary file 1 for details). For the calculation, a series of 500 snapshots taken from 25 ns of the total simulation, with intervals of 50 ps [111]. The following equation has been used to calculate the binding free energy [112,113,114]:

ΔG_bind_ = G_complex_ − (G_protien_ + G_ligand_) (e1),
ΔG_bind_ = ΔE_MM_ + ΔG_sol_ − TΔS (e2),
ΔE_MM_ = ΔE_elec_ + ΔE_vdW_ (e3),
ΔG_sol_ = ΔG_pol_ + ΔG_npol_ (e4),
ΔG_npol_ = γ × _SASA_ + b (e5), (4)

where, the total binding free energy is represented by ΔG_bind_, the energies of protein–ligand complex, protein (SULT1A1) and the ligand, PNP were depicted by G_complex_, G_protein_, and G_ligand_, respectively. Binding free energy can also be calculated using the equations e2–e5, where ΔG_sol_ denoted as the solvation energy, ΔG_pol_ and ΔG_npol_ represents polar and nonpolar solvation energy components, respectively. The electrostatic interaction energy was represented by ΔE_elec_ and van der Waals interaction energy represented by ΔE_vdW_. In equation (e5) γ and b are the experimental parameters, SASA stands for the solvent accessible surface area. Polar solvation effects have been computed by standard Poisson–Boltzmann equation, whereas SASA was used to calculate non-polar solvation energies through the following values γ (0.0226778 kJ.mol^−1^·Å^−2^) and b (3.84928 kJ.mol^−1^) [115].

## 5. Conclusions

The present study addressed the alteration of structural properties of SULT1A1 due to the R213H mutation, and also its effect on the substrates binding. A variety of structural characterization methods were applied followed by molecular dynamics simulations in this study, where the results highlighted significant structural differences in wild and mutant SULT1A1 proteins in terms of their native and ligand bounded states. Essentially, this mutation caused the actual conformational alteration in substrate binding loops, where the loop3 was mostly influenced. This manipulation changed the plasticity and intrinsic properties of the active site, which reduced not only the ligand flexibility in the pocket but also decreased the binding affinity, as well as the substrates recognition capabilities. Together with previous findings on SULT1A1 R213H, this study hereby contributes to a better understanding regarding the role of SULT1A1 mutation in cancer development.

## Figures and Tables

**Table 1:** Change of protein–ligand stability by means of MM-PBSA binding energy analysis, and the active site characteristics.

Systems	MM-PBSA ^$^ ∆G_binding_	CASTp ^&^		MM-PBSA ^#^ ∆G_binding_				
	(kJ/mol)	Volume	SASA	ΔE_vdW_ (kJ/mol)	ΔE_elec_ (kJ/mol)	ΔG_pol_ (kJ/mol)	ΔG_nonpo_ (kJ/mol)	ΔG_Binding_ (kJ/mol)
Wild		453.31	810.58	−	−	−	−	−
R213H		596.88	1020.92	−	−	−	−	−
Wild-PNP	110 ± 1.03	336.87	537.45	−80.18 ± 0.27	−8.23± 0.54	77.80 ± 0.36	−89.84 ± 0.30	−120.42 ± 0.68
R213H-PNP	60 ± 2.46	236.66	607.83	−73.25 ± 0.27	−24.06 ± 0.54	66.27 ± 0.56	−83.99 ± 0.34	−115.00 ± 0.62

^$^ Calculated based on the trajectories obtained 100 ns molecular dynamics simulation by YASARA dynamics software using default MM-PBSA binding energy calculation protocol. ^&^ Calculated by CASTpver 3.0 server (http://sts.bioe.uic.edu/castp/index.html). ^#^ Calculated based on the trajectories obtained 25 ns molecular dynamics simulation by GROMACS software using the g_mmpbsa tool.

## Supplementary Materials

Supplementary Materials can be found at https://www.mdpi.com/1422-0067/20/24/6256/s1.
