# Supplementary material for: Structural and Dynamic Characterizations Highlight the Deleterious Role of SULT1A1 R213H Polymorphism in Substrate Binding
Source: Int J Mol Sci. 2019 Dec 11;20(24):6256. doi: 10.3390/ijms20246256 (PMC6969939; doi:10.3390/ijms20246256)
Supplement: Supplementary file 1 [file ijms-20-06256-s001.pdf]

Supplementary Material and Methods

Simulation Method in "per Residue Energy Decomposition Analysis"

In order to calculate per residue energy decomposition analysis, additional independent MD simulations were carried out using GROMACS-4.4.5 [1]. The lowest energy structures of the protein–ligand complex, derived from the free energy landscape analysis in both wild and mutated type, were subjected for the simulation. The complexes were solvated in a dodecahedron periodic box with TIP3P water molecules at 10 Å marginal radius [2]. Afterwards, each system was neutralized by adding counter ions. The ionic stability of the system was adopted by adding NaCl (0.150 M). The steepest descent algorithm was then used to minimize the energy of all systems. After that, a simulation with constant volume was run for 200 ps, where the system was heated to 300 K. Following that, 500 ps NPT simulation with 2 fs time step was further run to equilibrate the pressure to 1 atm. During both simulations, the position of heavy atoms was restrained with the force constant of 1000 kJ/(mol nm^2), and the pressure and temperature were maintained by using the Berendsen algorithm [3]. Finally, 25 ns simulation was performed for each system at 300 K temperature and 1 atm pressure followed by v-rescaling [4] Parrinello–Rahman [5] pressure coupling method, respectively. The time step was maintained to 2 fs, and time constant has been kept at 0.1 and 1 ps for the temperature and pressure coupling, correspondingly. The non-bonded interactions within the short range were determined using cut off 1 nm, whereas long-range electrostatic interactions have been described by using the particle-mash-Ewald summation method with 1.2 Å grid spacing [6]. All bonds were constrained using the parallel LINCS method [7,8].

Supplementary Tables

Table S1. The conformational dynamics of SULT1A1 in both wild and mutated form by means of RMSD, Rg, SASA, and H-bond analysis. In each parameter, the average mean value is represented with standard error.

**Systems**	**RMSD (Å)**	**Rg (Å)**	**H-bond**	**SASA (nm^2)**
Wild	1.155 ± 0.001	18.34 ± 0.001	62.38 ± 0.101	138.76 ± 0.359
Wild-PNP	0.996 ± 0.001	18.26 ± 0.001	64.09 ± 0.105	135.32 ± 0.367
R213H	1.474 ± 0.002	18.35 ± 0.001	62.17 ± 0.100	139.01 ± 0.390
R213H-PNP	1.210 ± 0.003	18.31 ± 0.001	65.26 ± 0.103	136.79 ± 0.325

The results of statistical analysis were expressed as mean ± SEM, where SEM means the standard error of the mean. The calculation was done by using SPSS statistical SPSS v19 software, based on the 4000 snapshots obtained from the simulation trajectory.

Supplementary Figures

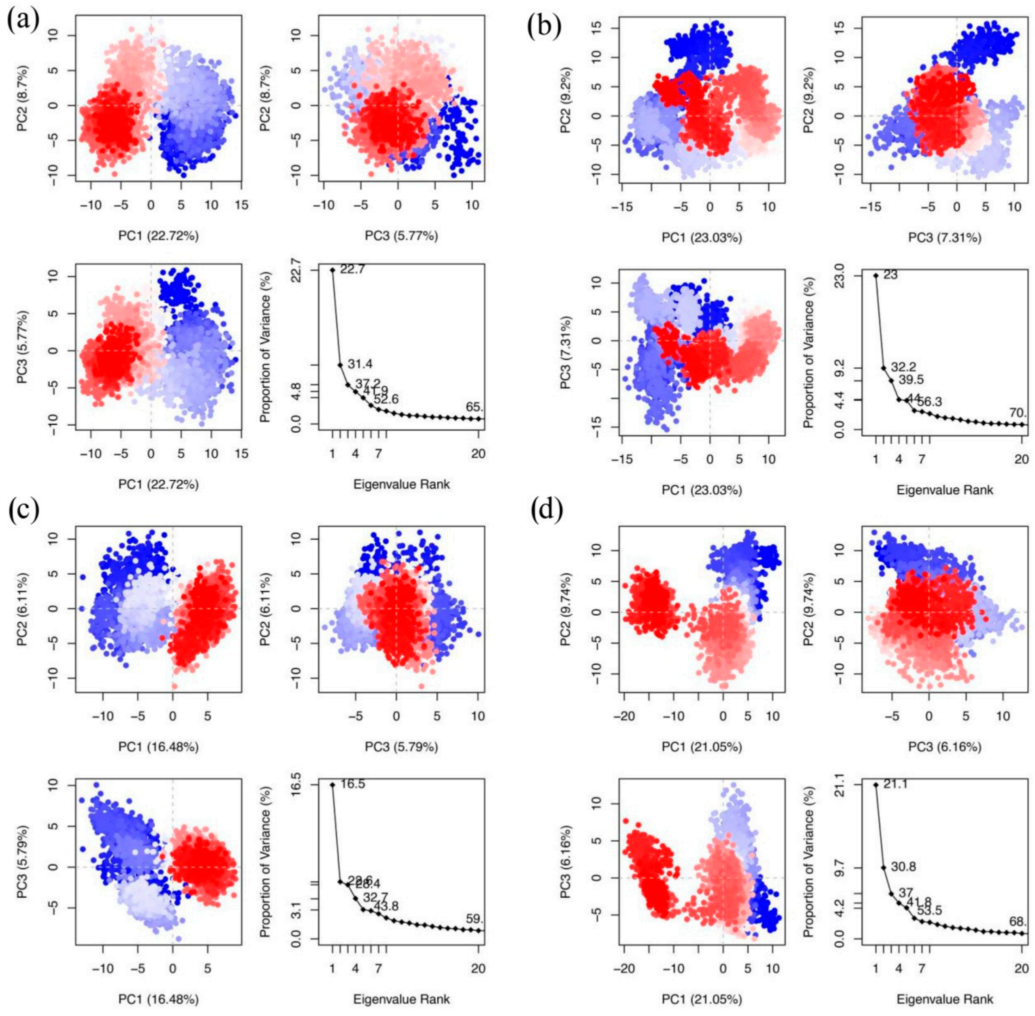

Figure S1. Principle Component Analysis (PCA) analysis for Wild (**a**), R213H (**b**), PNP-Wild (**c**), and PNP-R213H (**d**). In the figure, the projections of the simulated trajectories of SULT1A1 on first three eigenvectors. Throughout the *x* and *y* axes, each dot denotes the one conformation of the protein. The spread of blue and red color dots described the degree of conformational changes in the simulation, where color scale from blue to white to red is equivalent to simulation time. The blue indicates initial timestep, white is intermediate and final timestep is represented by red color.

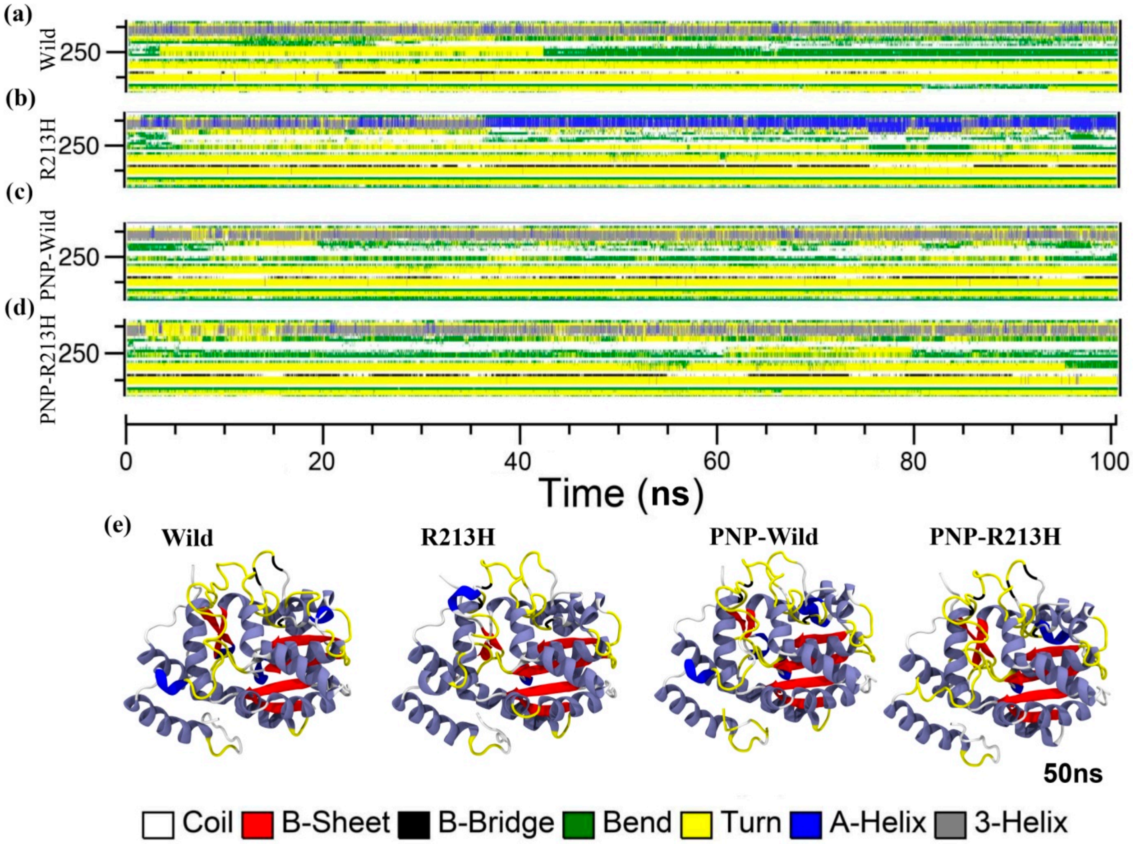

Figure S2. Secondary structural elements of loop3 residues (residue, 235–263) of wild protein (**a**), R213H (**b**), PNP-Wild (**c**), and PNP-R213H (**d**) during the molecular dynamics simulations. The bottom panel (**e**) represents the snapshot structures of different simulation systems, extracted from the individual trajectory at 50 ns timescale, in which the secondary structure is according to DSSP analysis.

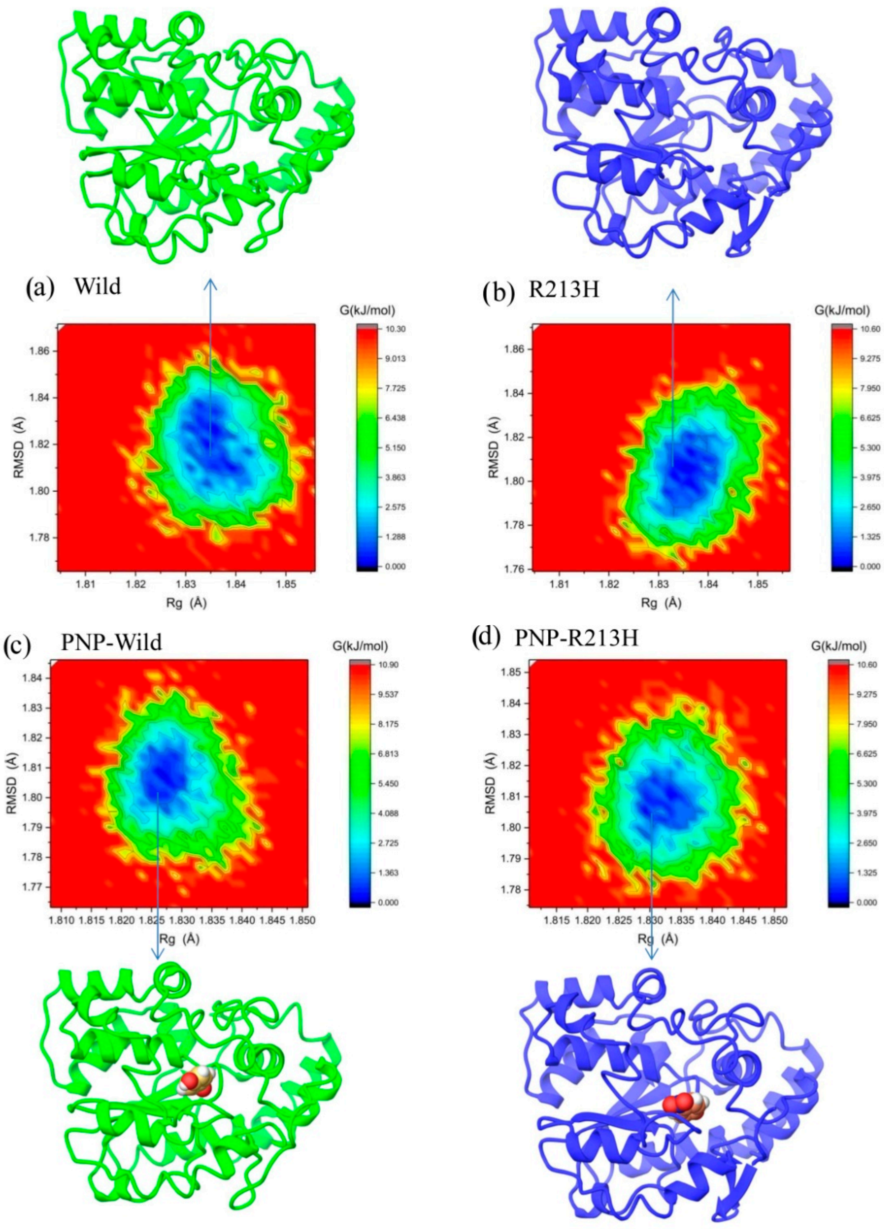

Figure S3. Trajectories explored in each system are projected on the free energy landscapes using as reaction coordinates the projection of each trajectory along the RMSD and second Rg for wild (**a**), R213H (**b**), PNP-Wild (**c**), and PNP-R213H (**d**) systems. The deep blue circles show the free energy minima basins in the landscape, corresponding to the most representative stable structures throughout the trajectories. The most favorable structure with global energy minima is represented at the right side of each panel.

References

1. Pronk, S.; Páll, S.; Schulz, R.; Larsson, P.; Bjelkmar, P.; Apostolov, R.; Shirts, M.R.; Smith, J.C.; Kasson, P.M.; van der Spoel, D.; et al. GROMACS 4.5: A high-throughput and highly parallel open source molecular simulation toolkit. *Bioinformatics* **2013**, *29*, 845–854.
2. Jorgensen, W.L.; Chandrasekhar, J.; Madura,J.D. Comparison of simple potential functions for simulating liquid water. *J. Chem. Phys.* **1983**, *79*, 926–935.
3. Berendsen, H.J.; Postma, J.P.M.; van Gunsteren, W.F.; DiNola, A.; Haak,J.R. Molecular dynamics with coupling to an external bath. *J. Chem. Phys.* **1984**, *81*, 3684–3690.

4. Bussi, G.; Donadio, D.; Parrinello, M.; Canonical sampling through velocity rescaling. *J. Chem. Phys*. **2007**, *126*, 014101.
5. Nosé, S.; Klein, M. Constant pressure molecular dynamics for molecular systems. *Mol. Phys.* **1983**, *50*, 1055–1076.
6. Darden, T.; York, D.; Pedersen, L. Particle mesh Ewald: An N· log (N) method for Ewald sums in large systems. *J. Chem. Phys*. **1993**, *98*, 10089–10092.
7. Hess, B. P-LINCS: A parallel linear constraint solver for molecular simulation*. J. Chem. Theory Comput*. **2008**, *4*, 116–122.
8. Hess, B., Bekke, H.; Berendsen, H.J.C.; Johannes, G.E.M.; Fraaije, J.G.E.M. LINCS: A linear constraint solver for molecular simulations. *J. Comput. Chem.* **1997**, *18*, 1463–1472.
